# Anisotropic Mechanical Response and Strain Localization of a Metallic Glassy-Fiber-Reinforced Polyethylene Terephthalate Fabric

**DOI:** 10.3390/ma14195619

**Published:** 2021-09-27

**Authors:** Jie Li, Bo Huang, Jun Shen, Jun Yi, Yandong Jia, Rongjie Xue, Gang Wang

**Affiliations:** 1 Laboratory for Microstructures, Institute of Materials, Shanghai University, Shanghai 200444, China; jli@shu.edu.cn (J.L.); jxy305@gmail.com (J.Y.); yandongjia@shu.edu.cn (Y.J.); g.wang@i.shu.edu.cn (G.W.); 2 College of Mechatronics and Control Engineering, Shenzhen University, Shenzhen 518060, China; junshen@szu.edu.cn; 3 School of Materials Engineering, Jiangsu University of Technology, Changzhou 213001, China; xuerongjie@jsut.edu.cn

**Keywords:** strain localization, metallic glassy fibers, digital image correlation, structural anisotropy

## Abstract

Optimizing the mechanical properties of composites through microstructural design has been a long-standing issue in materials science. In this study, we reinforced a typical polymer, i.e., polyethylene-terephthalate-woven fabric, with a type of Fe-based metallic glassy fiber (MGF) with an extremely large Young’s moduli. The MGF-reinforced fabrics, with three different fiber bundle orientations (0°, 45°, and 90°), were investigated by in situ electron-microscopy mechanical testing techniques in conjunction with a digital image correlation (DIC) technique. The fabrics exhibited a pronounced anisotropic mechanical response, and the associated characteristics were verified to depend on the fiber bundle orientation relative to the external load. Furthermore, localized strains near the intersections of the fiber bundles were found to be much higher than the global strain. It is confirmed that the restriction from warp to weft is the dominant factor influencing strain localization during deformation. Our results are enlightening for understanding the fracture mechanisms of composites.

## 1. Introduction

Fabric composites are widely used as structural materials in our daily life and in other fields, such as the automobile and aero industries [1,2]. Their mechanical properties are one of the major concerns of scientific research. The application of fibric composites requires more a refined analysis that takes into account multiaxial failure behavior [3,4,5]. Existing studies provide different perspectives for understanding the influence of the fabric architecture and loading direction on the mechanical properties, and the damage behaviors of woven fabric composites [6,7,8,9,10,11,12]. Cai et al. [6,7] investigated the tensile properties of unidirectional and woven fabric glass/epoxy composites under on- and off-axis loading and found that the Tsai-Wu failure criterion is more accurate with a modified interaction coefficient under multiaxial stress conditions compared with the Tsai-Hill, Hoffman and Yeh-Stratton criteria. Zhou et al. [8] studied the damage evolutions of woven fabric composites with three different fabric architectures by monotonic and cyclic on- and off-axis tension tests. The results show that compacted yarns and the lower crimp ratio lead to less damage at the same loading strain and abrupt rupture. Guo et al. [9] revealed that bias yarns significantly enhance the in-plane shear modulus and strength of the multiaxial angle-interlocked woven composites, with the primary failure modes identified as fiber slippage, tow splitting, and interfacial debonding. Koohbor et al. [10] extracted carbon-fiber-reinforced composites at different off-axis angles, tested them in uniaxial tension, and correlated globally applied stress and locally developed deformation in the materials. Zhai et al. [11] developed a coupled damage-plasticity model that describes the nonlinear off-axis tensile stress-strain relation of quasi-unidirectional E-glass fabric-reinforced polypropylene composites. Lu et al. [12] analyzed the on-axis uniaxial tensile behaviors and the tensile strengths of 2.5D woven fabric composites at different loading directions using a multiscale progressive damage finite element analysis simulating method. In spite of the extensive studies mentioned above, quantitative knowledge on the fracture mechanism of on- and off-axis loading, especially the local restraint effect relating to the microstructure, is still not fully established. Therefore, it is valuable to carry out more in situ analyses on the evolution of local strains before failure.

Apart from the uncertainty about the fracture mechanism, the mechanical properties (especially the strength) of fabric composites, mostly made up of polymers, also need to be further improved. With long-range disordered metallic bonding, metallic glasses (MGs) usually exhibit high strength and large toughness [13,14,15]. When the specimen is sufficiently small, it is difficult for the heterogenous nucleation of SBs, and the elastic limit and yielding strength of MGs increase [16,17]. An apparent small-size effect on compressive or tensile properties appears when the diameters of MG rods or fibers reach hundreds of nanometers [18,19]. With respect to the low dimensionality and excellent properties, it is intriguing to use metallic glass fibers (MGFs) as fillers to improve the mechanical performance of composites [20,21,22]. In addition, magnetically soft MGFs show giant magnetoimpedance (GMI) [23,24,25] and apparent permeability relaxation under electromagnetic excitations [26,27,28]. The MGF-reinforced composites can potentially be used as functional materials, such as electromagnetic interference filters and microwave absorbers [26,27,28].

Apart from the stress-strain curves and the fracture strength, other behaviors, such as local strain distribution and damage evolution, are also important for analysis and for modeling the anisotropic deformation and fracture behaviors of fabric composites. However, classical electrical resistance strain gauges do not have the adequate spatial resolution for the inhomogeneous local strain distributions. Digital image correlation (DIC), which has adequate spatial resolution and a full-field strain measurement, can be applied to determine local strain profiles, such as the maximum and minimum strain distribution [29,30,31]. The DIC technique has advantages in differentiating the slight variations of strain concentrations initiating damage caused by the local anisotropic microstructures. The main process of DIC technology is as follows: In order to capture the surface strain distributions during mechanical tests, artificial speckles were prepared on the surface of the specimen before loading. Then, several images were captured during the loading process by an image recording system, such as a CCD camera, optical microscope, or SEM. Finally, the local strains were computed through the correlation between these images and the initial image of the unloaded specimen using the software. The DIC technique has been applied to investigate the strain development and localization in, for example, anisotropic woven [32,33,34], laminate [35,36], and fiber- [37,38] and particle-reinforced composites [39,40].

In this paper, we explore the tensile properties of a polyethylene terephthalate fabric reinforced by soft magnetic Fe-based MGFs with structural anisotropy. Firstly, the tensile properties of the fabric specimens with three different loading directions are studied. Then, the strain-evolution processes are quantitatively analyzed with DIC technology. Finally, the restraint of warp on weft and the failure mechanisms of the fabric is discussed in detail.

## 2. Materials and Experimental Methods

**Figure 1:**
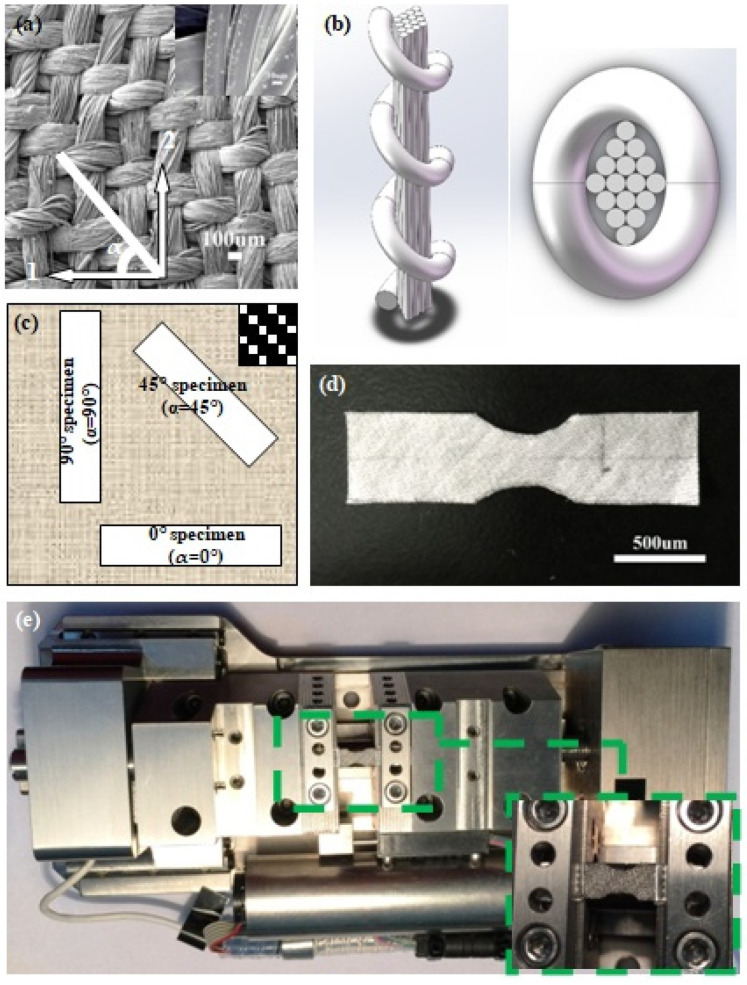
(**a**) Structure of the fabric, with the principal directions marked as 1 and 2. The image was captured by SEM, and the inset shows the detail of polyester fibers and an MGF; (**b**) A schematic of the bundle structure in 3D view and an overhead view; the orientation of the tensile specimens extracted from the original sheet is shown in (**c**), and the inset depicts the 1/2 twill fabric weave patterns; (**d**) A specimen used in the tensile experiment; (**e**) Miniature tension test frame with a composite specimen mounted.

The material under investigation in this work is an MGF-reinforced polyethylene terephthalate fabric, containing approximately 4% vol Fe_45.97_Co_19.06_Si_8.30_B_15.23_Ni_11.43_ MGFs. The production of the MGFs was achieved through the glass-coated melt spinning method, and the glassy cover was removed using an aqueous HF solution [41]. The diameters of both the warp and weft bundles of the fabric were approximately 140 µm, and each fiber bundle contained one MGF and 16 polyester fibers (Figure 1a). The averge diameter of the MGF was ~40 µm, and those of the fabric fibers were approximately 15 µm (see the inset of Figure 1a). A schematic of the bundle structure in 3D view, and an overhead view, are shown in Figure 1b. It can be seen that a bundle of polyester fibers is surrounded by one MGF. The fabric weave pattern used in the experiment is 1/2 twill weave (shown in the inset of Figure 1c). For the tensile test, specimens with a dog-bone shape (Figure 1d) were cut from the original fabric in the 0°, 45°, and 90° direction with fiber bundle orientation angles. The schematic view is shown in Figure 1c. The tensile specimen is provided as Figure 1d, with guidelines for the dimensions of the specimen of the ASTM Standard E8 (2004). At least three specimens with the same fiber bundle orientation were prepared and tested to ensure the reproducibility of the test results.

The mechanical properties of MGF and polyester fiber were conducted by a loading stage (Gata Microtest Series) (shown in Figure 1e) with a gauge length of 10 mm and a strain rate of 1.7 × 10^−4^ s^−1^. To avoid slip during the tension test, the polymer fibers were bonded to a paper frame with a rhombic hole [42], and the MGF was enhanced by Ni electrodeposition at both ends [43].

A thin-layer gold film was deposited on the specimen surface via magnetron sputter coating to visualize the strain fields by DIC technology. Then, the composite specimens were mounted on a tensile test device for in situ deformation in a Apollo 300 SEM (CamScan, Nottingham, UK). The loading stage (Gata Microtest Series), which is also used in tension tests on MGFs and polyester fibers, applied either by motor-driven or manually driven gears with a maximum tensile load of 2000 N, was installed inside the SEM chamber. Because there was no CCD conjunct with this scanning electron microscope, SEM images of the specimens were acquired in situ after each step of loading, and the digital speckle correlation images were acquired.

The load was applied monotonically at a rate of 0.4 mm min^−1^, which corresponds to a strain rate of 6.7 × 10^−4^ s^−1^, using the displacement control mode. The elongation of composite at rupture is much higher than that of fibers, so we used a higher strain rate for the composite tension experiment than that of fibers for testing convenience. A grip length of 5 mm was marked at each end of the specimens, and four aluminum tabs were attached to the grip area at both ends of the specimens using an epoxy adhesive to minimize stress concentrations and the possible damage caused by the serrated steel grips of the tensile machine. During loading, consecutive surface images were recorded with a 1280 × 1024-pixel array, and a pixel length of approximately 0.43 µm/pixel. A 540 × 540 pixel^2^ (1.27 × 1.27 mm^2^)-calculated domain was located in the middle of the specimen. An image was obtained prior to loading, which served as the reference (undeformed) record. Sequential images were then analyzed with respect to this reference image using DIC, and the strain distributions at the different loads were then identified by a series of pronounced contour maps.

## 3. Results and Discussion

**Figure 2:**
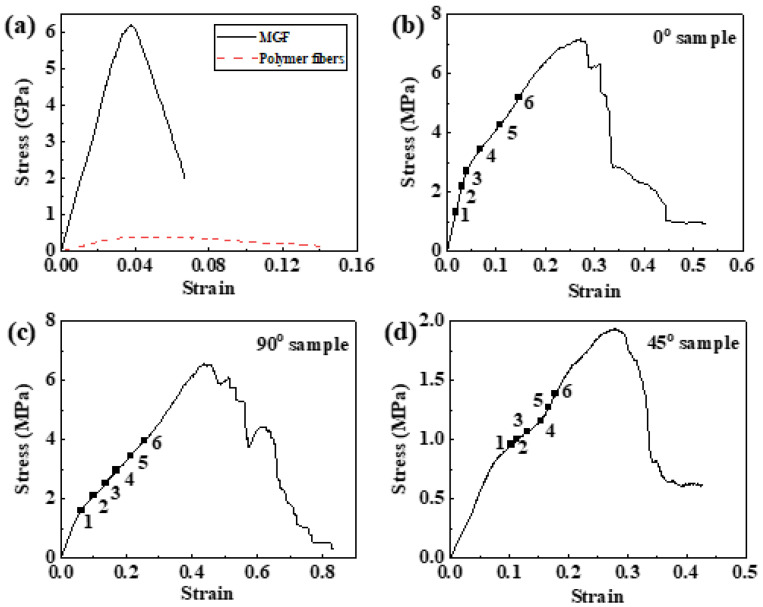
Tensile stress-strain curves of (**a**) MGF and polymer fiber, (**b**) 0° specimen, (**c**) 90° specimen, and (**d**) 45° specimen.

Uniaxial tension tests were carried out to examine the mechanical strength and elastic deformation of the fibers. Figure 2a shows the tension stress-strain curves of the MGF and polymer fiber. The deformation processes of the MGF and polymer fiber are similar and can be divided into two stages: elastic deformation and plastic deformation. The tension test results of MGF and polymer fibers are shown in Table 1. The elastic limit, the yield stress, and the elastic modulus of the MGF are 2.8%, 5.22 GPa, and 187 GPa, respectively, whereas the polymer fiber exhibits an elastic strain limit of 2.6% at a yield stress of 0.32 GPa, and the calculated elastic modulus is 12.34 GPa (shown in Table 1). The mixing rule [44] can be written as $\sigma_{m}^{\alpha }=V_{p}\sigma_{p}^{\alpha }+V_{m}\sigma_{m}^{\alpha }$, where $\sigma_{m}^{\alpha }$, $\sigma_{p}^{\alpha }$, and $\sigma_{m}^{\alpha }$ are the mechanical parameters of the composite, polyester fiber, and MGF, respectively, and $V_{p}$ and $V_{m}$ are the volume fraction of polyster fiber and MGF, respectively. In this fabric, $V_{p}$ is 69%, and $V_{m}$ is 31%. Compared with the polymer fiber, the MGF has almost the same elastic strain limit. However, the MGF exhibited a much higher yield stress and the elastic strain modulus, which enhances the mechanical properties of the fabric.

The tension stress-strain curves of specimens with three different fiber bundle orientations (0°, 90° and 45°) are plotted in Figure 2b–d. The stress-strain plots show a linear trend at the initial part of the curve, and then a nonlinear feature appears until the fabric failure. A possible cause of this deviation from linearity is local damage initiation; the damage process will be discussed later in this paper. For both the 0° and 90° specimens, the fracture stress is approximately 7 MPa. By comparing the measured stress of 0° and 90° specimens, it can be clearly observed that the strain of 0° specimens is smaller than that of 90° specimens at identical stress, indicating a stronger deformation resistance in 0° specimens. The maximum stress for the 45° specimen is less than 2 MPa, which demonstrates that the 45° specimen possesses the least stiffness and ductility among the three specimens. These results suggest an anisotropic mechanical response, due to the strong sensitivity to the orientation of the fiber bundles. The elastic moduli *E_x_* of woven composites at different loading directions can be expressed as [45]

(1) $$E_{x}^{-1}=E_{1}^{-1}cos^{4}\theta +\left(G_{12}^{-1}-2v_{12}E_{1}^{-1}\right)sin^{2}\theta cos^{4}\theta +E_{2}^{-1}sin^{2}\theta \;$$

where *E*_1_ is longitudinal modulus, *E*_2_ is transverse modulus, *G*_12_ is shear modulus, *υ*_12_ is Poisson’s ratio, and *θ* is the off-axis loading direction. The failure-mode-independent and stress-based Tsai-Wu failure criterion [6,7] is applied to the off-axis tensile strength *σ_x_*, and has the form [32]

*F_xx_σ_x_*^2^ + *F_x_σ_x_* = 1, (2)

where $F_{xx}={\left(X_{t}X_{c}\right)}^{-1}cos^{4}\theta +{\left(Y_{t}Y_{c}\right)}^{-1}sin^{4}\theta +\left[S_{12}^{-2}-{\left(X_{t}X_{c}Y_{t}Y_{c}\right)}^{-\frac{1}{2}}\right]sin^{2}\theta cos^{2}\theta$ and $F_{x}=\left(X_{t}^{-1}-X_{c}^{-1}\right)cos^{2}\theta +\left(Y_{t}^{-1}-Y_{c}^{-1}\right)sin^{2}\theta$ with *X_t_*/*X_c_* is the uniaxial tensile/compressive strength in longitudinal direction, *Y_t_*/*Y_c_* is the uniaxial tensile/compressive strength in transverse direction, and *S*_12_ is the in-plane shear strength in the material coordinate system. As reported, a minimum value usually exists for *E_x_* or *σ_x_*, with *θ* equaling 45° based on Equation (1) or (2). The smallest stiffness and the fracture stress of the 45° specimen is consistent with the reported results.

**Figure 3:**
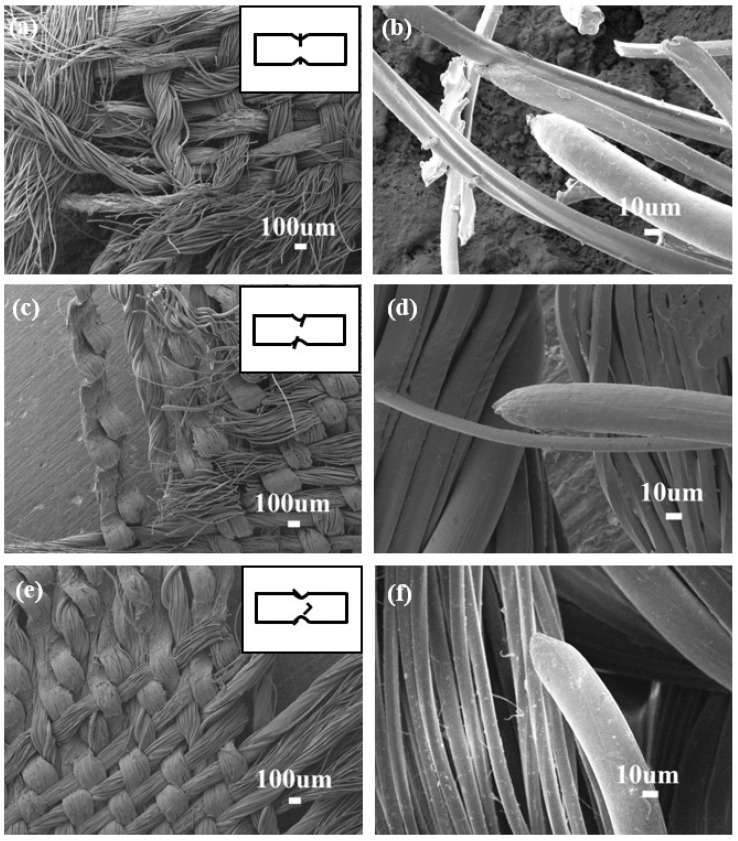
The SEM images of the fractured surface of (**a**) 0° specimen, (**c**) 90° specimen, and (**e**) 45° specimen with the insets showing the corresponding whole fractured surface schematically by the dashed lines. Lateral surface of the fractured MGFs for (**b**) 0° specimen, (**d**) 90° specimen, and (**f**) 45° specimen.

To explore the underlying failure mechanism microscopically, the corresponding specimens were investigated by SEM and the results are shown in Figure 3. For the 0° specimen, the fracture surface is almost perpendicular to the tension direction (as illustrated by the dashed line in the inset of Figure 3a). The fracture surface is an inclined plane for the 90° specimen (as illustrated in the inset of Figure 3c). In contrast, the 45° specimen is fractured at ±45° angled planes (as illustrated in the inset of Figure 3e). The SEM images of 0° and 90° specimens, randomly selected from the fracture surfaces, present similar behaviors, which both fracture throughout the specimen cross-section (Figure 3a,c). The straight fracture edges may be caused by the fiber pull-out mechanism. The SEM image of the 45° specimen, selected from the 45° angled plane of the conical fracture surface, provides evidence of large shear deformation (Figure 3e). Additionally, the MGFs exhibit apparent necking after failure (Figure 3b,d,f).

**Figure 4:**
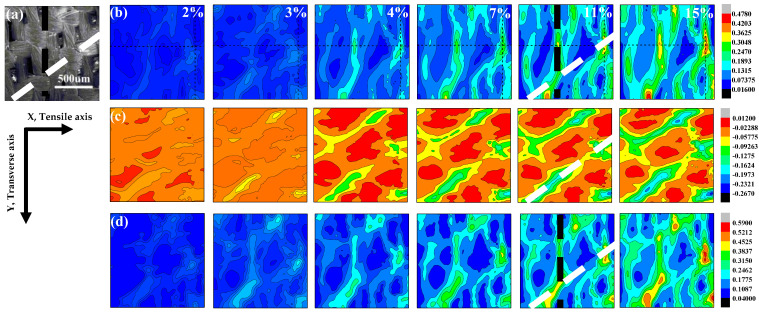
DIC images of strained 0° specimen: (**a**) speckle image of the 0° specimen before loading; (**b**) contour maps of the *ε_xmax_* field; (**c**) contour maps of the *ε_ymax_* field; (**d**) contour maps of the *γ_max_* field.

In order to explore the underlying mechanism of the two different failure modes under tension, we analyze the evolution of local strain fields obtained by the in situ DIC measurement. The SEM images of the 0° specimens before loading are shown in Figure 4a. On the basis of the undeformed state, strain-field development, including the *ε_x_* fields along the loading direction (*x*-axis), the *ε_y_* fields perpendicular to the loading direction (*y*-axis), and the shear-strain (*γ_xy_*) fields, can be calculated. If the coordinate rotates by an arbitrary angle of *θ*, the local stresses can be expressed as [46]

(3) $$\varepsilon_{x}^{\prime }=\frac{1}{2}\left[\left(\varepsilon_{x}+\varepsilon_{y}\right)+\left(\varepsilon_{x}-\varepsilon_{y}\right)\cos2\theta +\gamma_{xy}\sin2\theta \right],$$

(4) $$\varepsilon_{y}^{\prime }=\frac{1}{2}\left[\left(\varepsilon_{x}+\varepsilon_{y}\right)-\left(\varepsilon_{x}-\varepsilon_{y}\right)\cos2\theta -\gamma_{xy}\sin2\theta \right],$$

(5) $$\gamma_{xy}^{\prime }=\left(\varepsilon_{x}-\varepsilon_{y}\right)\sin2\theta +\gamma_{xy}\cos2\theta .$$

From Equations (3)–(5), the express of the Mohr circle can be deduced as

(6) $${\left[\varepsilon_{x\left(y\right)}^{\prime }-\frac{1}{2}\left(\varepsilon_{x}+\varepsilon_{y}\right)\right]}^{2}+{\left(\frac{\gamma_{xy}^{\prime }}{2}\right)}^{2}=\frac{1}{4}\left[{\left(\varepsilon_{x}-\varepsilon_{y}\right)}^{2}+\gamma_{xy}^{2}\right].$$

From Equations (3)–(6), the local principle linear strains and corresponding rotation angles can be easily calculated as

(7) $$\varepsilon_{x\left(y\right)max}=\frac{1}{2}[\left(\varepsilon_{x}+\varepsilon_{y}\right)\pm \sqrt{{\left(\varepsilon_{x}-\varepsilon_{y}\right)}^{2}+\gamma_{xy}^{2}}],$$

(8) $$\theta_{xmax}=\frac{1}{2}arctan\left(\frac{\gamma_{xy}}{\varepsilon_{x}-\varepsilon_{y}}\right),$$

(9) $$\theta_{ymax}=\frac{1}{2}\arctan\left(\frac{\gamma_{xy}}{\varepsilon_{x}-\varepsilon_{y}}\right)+\frac{\pi }{2}.$$

The local principle shear strain and rotation angle can be determined as

(10) $$\theta_{ymax}=\frac{1}{2}\arctan\left(\frac{\gamma_{xy}}{\varepsilon_{x}-\varepsilon_{y}}\right)+\frac{\pi }{2},$$

(11) $$\theta_{ymax\left(min\right)}=\frac{1}{2}\arctan\left(\frac{\varepsilon_{x}-\varepsilon_{y}}{\gamma_{xy}}\right)=\theta_{xmax}+\frac{\pi }{2}.$$

Therefore, the maximum local normal strains *ε_xmax_* and *ε_ymax_*, and the maximum local shear strain *γ_max_*, can be calculated by Equations (7)–(11).

Figure 4b–d display the strain developments for the *ε_xmax_*, *ε_ymax_*, and *γ_max_* of the 0° sample, with global tensile strains as 2%, 3%, 4%, 7%, 11%, and 15%, respectively. It can be observed that *ε_xmax_* and *γ_max_* both increase with the global strain. In the initial stage, when the global strain is less than 3%, there is no obvious stain concentration zone in the contour maps. In Figure 2b, the stress and strain are linearly related. When the global strain is more than 7%, the strain concentration zone becomes more and more obvious, and the stress has a nonlinear relation with the strain, as shown in Figure 2b. Furthermore, large strain-concentration striations are formed in the *ε_xmax_* and *γ_max_* strain fields that lie at an angle of ~90° or ~35° to the direction of the tension load (marked by the black and white dashed lines in Figure 4b,d). Moreover, *ε_ymax_* decreases with the global strain, and the local strain striations of small *ε_ymax_* lie at an angle of ~35° to the direction of the tension load (marked by the white dashed line in Figure 4c). Compared with the SEM image in Figure 4a, it can be observed that the local large *ε_xmax_* and *γ_max_* striations locate at the warp and weft intersections or along the boundaries of the warp bundles, while the *ε_ymax_* striations locate only at the warp and weft intersections.

**Figure 5:**
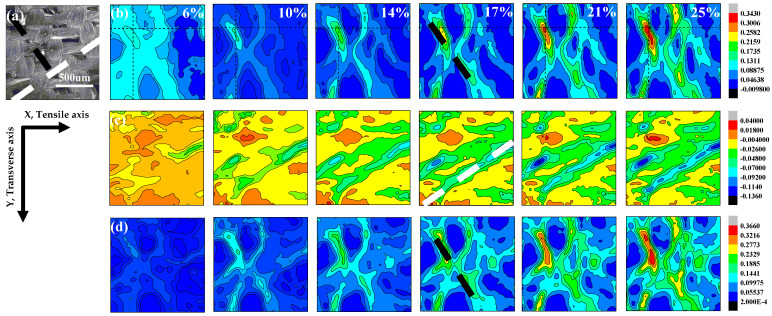
DIC images of strained 90° specimen: (**a**) Speckle image of the 90° specimen before loading. (**b**) Contour maps of the *ε_xmax_* field. (**c**) contour maps of the *ε_ymax_* field. (**d**) contour maps of the *γ_max_* field.

The SEM image of the 90° specimen before deformation is mapped in Figure 5a. Figure 5b–d show the strain developments for *ε_xmax_*, *ε_ymax_*, and *γ_max_* of the 90° sample, with global tensile strains as 6%, 10%, 14%, 17%, 21%, and 25%, respectively. It is can be seen that the global tensile strain values for the 90° specimen are different from the 0° specimen. The elongation at maximum force for the 0° specimen is less than 0.3, but for the 90° specimen, the elongation at maximum force is more than 0.4, as shown in Figure 2b,c. In order to obtain the whole evolution process of the strain concentration, different global tensile strain values are chosen for the 90° specimen. *ε_xmax_* and *γ_max_* both increase with the global strain, and the strain concentration zone becomes more and more obvious. In addition, the stress has a nonlinear relation with strain accompanied by strain concentrating, shown in Figure 2c. It seems that the large local *ε_xmax_* and *γ_max_* striations all locate at the middle of the warp and weft intersections (as marked by the dashed black line in Figure 5a,b,d). It is interesting that the *ε_ymax_* striations lie at an angle of ~35° to the direction of the tension load (marked by the white dashed line in Figure 5c).

**Figure 6:**
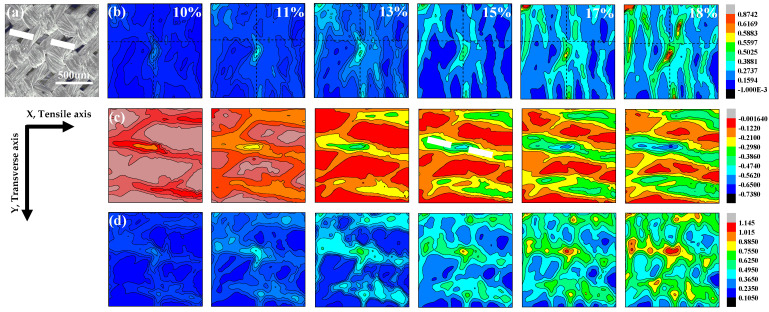
DIC images of strained 45° specimen. (**a**) Speckle image of the 45° specimen before loading. (**b**) Contour maps of the *ε_xmax_* field. (**c**) Contour maps of the *ε_ymax_* field. (**d**) Contour maps of the *γ_max_* field.

Similarly, the SEM image of the 45° specimen before deformation is illustrated in Figure 6a. Figure 6b–d show the strain developments for *ε_xmax_*, *ε_ymax_*, and *γ_max_* of the 45° sample, with the global tensile strains as 10%, 11%, 13%, 15%, 17%, and 18%, respectively. The *ε_xmax_* and *γ_max_* concentrations are both isolated in the center of the specimen, and there are many overlapping regions of the two strain-concentration fields. With the emergence of strain concentration regions, the stress shows a nonlinear relation with strain (Figure 2d). However, the strain-concentration striations are observed in the *ε_ymax_* field at an angle of 12° to the direction of the tension load, which is fitted to the line of the adjacent warp and weft intersections (marked as a white dashed line in Figure 6a,c).

**Figure 7:**
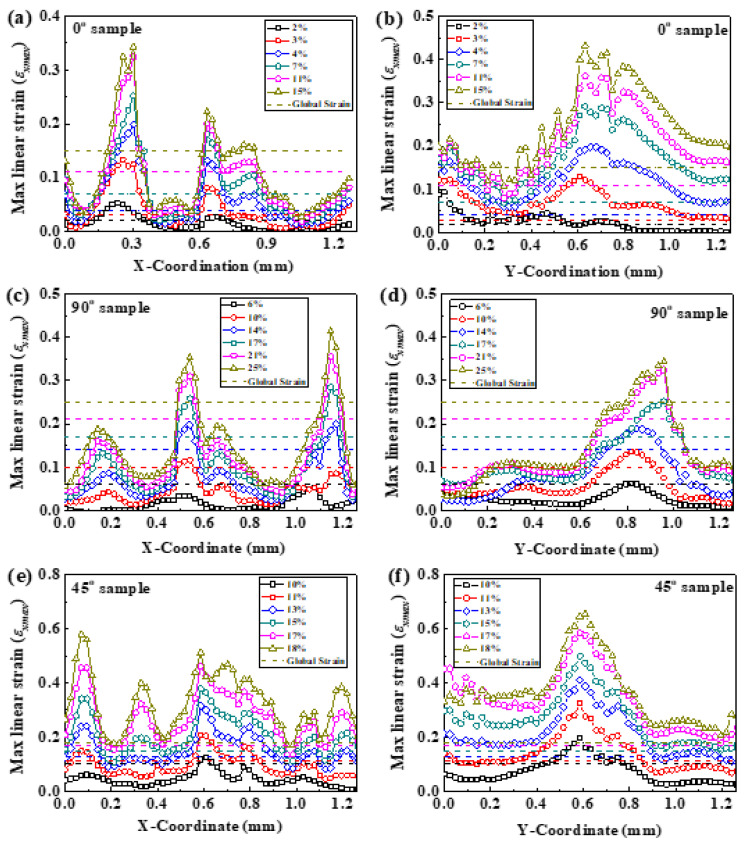
Profile of the distributions of the maximum *ε_xmax_* values at different global strains of (**a**) 0°, (**c**) 90°, and (**e**) 45° specimens on the maximum shear stress plane, i.e., along the dash lines (*x*-axis), and at different global strains of (**b**) 0°, (**d**) 90°, and (**f**) 45° specimens along the dotted lines (*y*-axis) in Figure 4, Figure 5 and Figure 6.

To further characterize the development of strain localization with global strain, the distribution profiles of the *ε_xmax_* values at different global strains for the three specimens on the principal stress plane, i.e., along the fine dash lines (*x*-axis) and dot lines (*y*-axis) in Figure 4, Figure 5 and Figure 6, are displayed in Figure 7. For the 0° and 90° specimens, all of the *ε_xmax_* along the *x*-axis and *y*-axis increases when the global strain increases (Figure 7a–d). Moreover, the maxima and minima of the *ε_xmax_* periodically emerges along the *x*-axis (Figure 7a,c) when the global strain increases, but the maximum of *ε_xmax_* only presents at the middle along the *y*-axis (Figure 7b,d). The peaks in Figure 7 correspond to the strain-concentrated regions along the dashed lines in Figure 4b, Figure 5b, and Figure 6b, which locate around the warp and weft intersections. The fiber bundles are perpendicular, or parallel, to the load direction in both of the 0° and 90° specimens. When the tensile load increases, the cross-section of the specimen decreases gradually, which results in the restraint of warp on weft at the warp and weft intersections and leads to strain localization behaviors. For the 0° and 90° specimens, the fiber bundles parallel to the tensile axis are under an isostrain condition [39] at lower global strain. It is shown in Figure 7a,c that the local strain values along both the x- and y-axes seem homogeneous at lower global strains, and most of these local strains are smaller than the global strain. However, with increasing load, the fiber bundles at the end of the specimen (near the grids) cannot generate an equal tensile strain with that of the middle part of the specimen because of the restraint of warp on weft. Moreover, when the global strain becomes larger, local strains fluctuate more apparently. This result suggests that the restraint of the warp bundles on the weft bundles increases with the global strain (Figure 7a–d). With the heterogeneous strain accumulation, the strain-concentration striations emerge along the *x*-axis (Figure 4b and Figure 5b) where there are regions of periodic strain maxima or minima (Figure 7a,c). The different distances of periodic strain also reveal the different restraint effects of warp yarn on weft for the 0° and 90° specimens. Because of the striation distribution of the *ε_xmax_* and *γ_max_* fields, and the periodic strain maxima or minima along the *x*-axis, the nature of failure for both the 0° and 90° specimens can be explained by the fiber pull-out mechanism. Hence, the maximum stress of the 0° and 90° specimens are related to the number of transverse fiber bundles in the cross-section. Due to the same specimen dimensions and 1/2 twill woven pattern of the fabric, the two specimens have the same quantity of fiber bundles at the cross-sections and, thus, both the 0° and 90° specimens fracture at approximately 7 MPa (Figure 2b,c). Additionally, the weft gap of the 90° specimen, which lies between the adjacent warp and weft intersections, is longer than that of the 0° specimen, thus leading to a greater elongation of the 90° specimen (Figure 2b,c).

For the 45° specimen, the maxima and minima of *ε_xmax_* periodically emerge along the *x*-axis (Figure 7e), but the maximum of *ε_xmax_* only presents at the middle along the *y*-axis. The global strain increase in Figure 7f is similar to the above two specimens. In addition, all of *ε_xmax_* along the *x*-axis and *y*-axis increases when the global strain increases, shown in Figure 7e,f. However, the fiber bundles of the 45° specimen are neither parallel nor perpendicular to the tensile axis but are oriented at ±45° angles with respect to the tensile axis. Thus, the number of warp and weft intersections along the *x*-axis is more than that of the 0° and 90° specimens (Figure 4a, Figure 5a, and Figure 6a), leading to more peaks of *ε_xmax_* in Figure 7e related to the restraint effect. It is shown in Figure 7e,f that the local strain values along both the *x*- and *y*-axes seem homogeneous at lower global strains, and that most of these local strains are smaller than the global strain. However, with the tensile increasing, the cross-section of the specimen decreases, and the fiber bundles rotate towards the direction parallel to the load as the load increases gradually, which results in the restraint of warp on weft, and leads to strain localization behaviors. With the heterogeneous strain accumulation, the small separated strain-concentration regions emerge in the middle of the specimen (Figure 6d), leading to the maxima of *ε_xmax_* presenting in the middle along the *y*-axis in Figure 7f. Owing to the different load responses of the different components in the fabric, i.e., polyester fibers and MGFs, as well as the described fiber trellising phenomenon [47,48] under tension load, a shear strain would develop in the interfacial regions of different fiber bundles. Moreover, during the trellising shear deformation, the angle and interval between the weft and warp fiber bundles varies with the shear deformation. At an earlier stage of tension, smaller yarn gaps will introduce shear locking. However, with the increase in loading, the inter-yarn gap between the two adjacent parallel yarns becomes larger, which will make the fabric looser and will finally lead to a lower shear stiffness of the specimen. Therefore, the far-field load is shared between the polyester fibers and MGFs under a shear-type deformation with a significantly larger plastic deformation. For the fabric system studied here, this phenomenon becomes clear. High magnitudes of shear strain can result in damaging accumulation by void coalescence and result in an initial fracture in the center of the specimen [49]. In Figure 6d, the maximum of *γ_max_* is isolated in the center of the specimen, and the specimen fails from that point. Thus, the 45° specimen exhibits a highly roughened conical fracture surface, as demonstrated in Figure 3e.

## 4. Conclusions

Based on in situ electron microscopy mechanical testing, in conjunction with DIC technology, the anisotropic mechanical response and strain localization of an MGF-reinforced fabric is investigated in this study. The following conclusions can be drawn:

(1) The MGF is attributed to the enhanced mechanical properties of the fabric. The deformation processes of the MGF and polymer fiber are similar and can be divided into the elastic stage and the plastic stage. Although they had almost the same elastic strain limit, the MGF exhibited a much higher yield stress in the elastic strain modulus compared with the polymer fiber. According to the mixing rule, the MGF is beneficial to the mechanical enhancement of fabric.
(2) The restraint of warp yarn on weft in the process of deformation is verified to be beneficial to the strain localization, which is the main cause of the fracture for fabric. With the local strain accumulation, the strain-concentration regions emerge. For the 0° and 90° specimens, the fiber bundles are parallel or perpendicular to the tensile axis. With the global strain increasing, the cross-section of the specimen decreases, resulting in the restraint of warp yarn on weft, and leading to strain localization behaviors. Moreover, the strain-concentration striations appear at the warp and weft intersections or along the boundaries of the warp bundles. For the 45° specimen, fiber bundles rotate towards the direction parallel to the load as the load increases gradually, which bring about the restraint effect and strain localization, and some small, isolated regions locate in the center of the 45° specimen.
(3) The orientation of the fiber bundles strongly affects the fracture mechanism of the fabric because of different local fracture mechanisms. For the 0° and 90° specimens, the strain-concentration striations expose the fiber pull-out mechanism with straight fracture edges, and the 45° specimen is confirmed to fracture by shearing with a rough conical fracture surface due to the small, separated strain-concentration regions and the fiber trellising effect.

Overall, this study provides novel insights into the restraint of warp yarn on weft during the deformation process and improves the understanding of the deformation process and fracture mechanisms of a twill fabric. Although these results only refer to a twill fabric, they still provide some contributions to the referred topic, for example, advancing the understanding of the synergetic effects that often arise in hybrid composites, and assisting in contributing to the safety and reliable design of composite structures.

## Figures and Tables

**Table 1:** The tension test results of the MGF and polymer fiber, i.e., Young’s modulus (*E*), elastic limit (*ε_e_*), yield stress (*σ_y_*), maximum tension stress (*σ_t_*), and plastic strain (*ε_p_*).

Sample	*E* (GPa)	*ε_e_*	*σ_y_* (MPa)	*σ_t_* (MPa)	*ε_p_*
MGF	186.5	0.03	5.2	6.2	0.04
Polymer fiber	12.3	0.03	0.3	0.4	0.1

## Data Availability

Data are available upon request to the corresponding author.
